# A Novel Supergene Controls Queen Size and Colony Social Organization in the Ant *Myrmica ruginodis*

**DOI:** 10.1093/molbev/msaf255

**Published:** 2025-10-13

**Authors:** Hanna Sigeman, Perttu Seppä, Philip A Downing, Matthew T Webster, Heikki Helanterä, Lumi Viljakainen

**Affiliations:** Ecology and Genetics Research Unit, University of Oulu, P.O. Box 3000, FI-90014 Oulu, Finland; Department of Medical Biochemistry and Microbiology, SciLifeLab, Uppsala University, P.O. Box 582, 751 23 Uppsala, Sweden; Organismal and Evolutionary Biology Research Programme, University of Helsinki, P.O. Box 65, 00014 Helsinki, Finland; Ecology and Genetics Research Unit, University of Oulu, P.O. Box 3000, FI-90014 Oulu, Finland; Department of Medical Biochemistry and Microbiology, SciLifeLab, Uppsala University, P.O. Box 582, 751 23 Uppsala, Sweden; Ecology and Genetics Research Unit, University of Oulu, P.O. Box 3000, FI-90014 Oulu, Finland; Tvärminne Zoological Station, University of Helsinki, J.A. Palménin tie 260, 10900 Hanko, Finland; Ecology and Genetics Research Unit, University of Oulu, P.O. Box 3000, FI-90014 Oulu, Finland

**Keywords:** supergene, Myrmica ruginodis, ant, polygyny

## Abstract

Large independently evolved supergenes control colony social organization and queen reproductive strategies in several ant lineages. Their independent origins, as well as the similarities of the associated phenotypes, make ant supergenes a promising system for studying the parallel evolution of genome organization and adaptability. However, the genetic basis of differences in social organization and queen phenotypes remains unknown in many ant species, limiting the potential power of this system for comparative studies. We investigated the genetic basis of colony social organization in the queen-size dimorphic ant *Myrmica ruginodis* by sampling 95 queens from 31 colonies in southern Finland. Whole-genome sequencing revealed a novel 9 Mb supergene associated with both queen size and social organization. Queens homozygous for the AA haplotype were larger and found only in single-queen colonies, while queens in multiple-queen colonies were smaller and carried only AB and BB genotypes. This supergene is not homologous to previously identified supergenes in ants, suggesting it arose through a distinct evolutionary pathway.

## Introduction

Supergenes get their name from their ability to control complex phenotypes while being inherited as single Mendelian units. As the genes that make up a supergene are clustered on the same chromosome and rarely segregate during recombination, they can evolve into stable haplotype polymorphisms encoding distinct phenotypes. Supergenes underlie many iconic examples of phenotypic variation in nature, including those related to alternative reproductive strategies. For example, they control male sneaker behavior in the ruff (Küpper et al. 2016; Lamichhaney et al. 2016), assortative mating and parental care in the white-throated sparrow (Tuttle et al. 2016), and exaggeration of secondary sexual traits in male dwarf spiders (Hendrickx et al. 2022). Although the diversity and functionality of supergene systems are increasingly well understood (see e.g. Gutiérrez-Valencia et al., (2021) for a recent review), our ability to investigate the genetic basis of parallel complex phenotypic evolution remains limited. To do so effectively, we need multiple independent origins of supergenes controlling the same trait, ideally within a single study system.

Ants are a promising system. In this group, alternative reproductive strategies often manifest as discrete queen phenotypes living in colonies with different social organization. The ancestral strategy involves a queen morph capable of dispersal and independent colony founding (Hölldobler and Wilson 1990). From this (*monogynous*) single-queen colony structure, queens have repeatedly evolved into philopatric, and often smaller, morphs living in (*polygynous*) multi-queen colonies. These polygynous queens often forego the risky life stage of independent dispersal, and instead become one of several reproductive queens in their natal colony (see e.g. Heinze and Tsuji 1995; Rüppell and Heinze 1999; Wolf and Seppä 2016). Although the exact variants controlling colony social organization have yet to be definitively identified, large haplotype blocks behaving like supergenes are associated with within-species differences in queen reproductive strategy and colony social organization in least 5 lineages: *Cataglyphis* (Lajmi et al. 2024), *Formica* (Purcell et al. 2014; Brelsford et al. 2020), *Leptothorax* (Braim 2015), *Pogonomyrmex* (Errbii et al. 2024), and *Solenopsis* (Wang et al. 2013). These supergenes appear to have evolved independently (Wang et al. 2013; Brelsford et al. 2020; Errbii et al. 2024; Lajmi et al. 2024), offering a growing framework for testing hypotheses on parallel supergene evolution and genomic flexibility. However, many ant species with multi-queen colonies (as compiled by e.g. (Boulay et al. 2014; Bell-Roberts et al. 2024)) remain genomically unstudied, suggesting that numerous supergenes are yet to be found. Detection of novel supergenes is therefore needed to fully harness the power of this system for comparative analyses.

We searched for a novel supergene controlling queen size and colony social organization in the common Palearctic red ant *Myrmica ruginodis* (Nylander). This species is characterized by queen-size dimorphism, which is linked to distinct reproductive strategies. Large *macrogyne* queens disperse from their natal colonies to join mating swarms and establish new colonies independently, where they are the sole reproductive female. Small *microgyne* queens are found in colonies where multiple queens coexist and reproduce. Microgynes tend to mate within or near their natal colonies to which they are often recruited back as reproductive queens, and new colonies are founded when old ones split (*budding*, Brian and Brian 1949, 1955). The association between queen morph and colony social organization in *M. ruginodis* is not absolute, however. Queen size tends to have a bimodal distribution and queens are on average larger in single-queen than in multi-queen colonies, but there is some size overlap. Moreover, while most macrogynes disperse and most microgynes are recruited back to their natal colony, queens of both types sometimes deviate from these patterns (Elmes 1991; Wolf et al. 2018).

Although the genetic mechanisms underlying queen size and colony social organization in *M. ruginodis* are currently unknown, previous genetic studies provide important background information for the system. Allozymes and DNA microsatellite data have revealed only weak genetic differentiation between monogynous and polygynous colonies (Wolf 2016), explained partly by males from both colony types joining mating swarms (Wolf and Seppä 2016). Most monogynous colonies are simple families, where workers are daughters of the resident queen. However, sometimes the resident queen is not the mother of all workers (Seppä 1994). Multiple matrilines within monogynous ant societies have also been found in other ant species, and can be explained by worker recruitment of a new queen in colonies where the previous queen has died (so-called *serial polygyny*; reviewed in Heinze and Keller 2000). High relatedness among queens in polygynous colonies confirms that most queens rejoin their natal colony after mating, but unrelated queens also occur in them (Seppä 1994; Walin and Seppä 2001; Wolf et al. 2018). Lastly, queen-size in *M. ruginodis* is a heritable trait and is expected to be fully or partially under genetic control (Wolf 2016).

Here we identify a novel 9 Mb supergene in *M. ruginodis* and show that it controls both queen-size and colony social organization. The supergene is non-homologous to previously identified and well-characterized supergenes controlling monogyny/polygyny in ants (*Cataglyphis* (Lajmi et al. 2024), *Formica* (Purcell et al. 2014), *Pogonomyrmex* (Errbii et al. 2024), and *Solenopsis* (Wang et al. 2013)), suggesting an independent origin within the *Myrmica* lineage. It is also non-homologous to a second supergene in *Formica*, so far detected only in *F. cinerea*, which controls queen size within polygynous colonies (Scarparo et al. 2023). Our discovery solves the long-standing mystery of alternative reproductive strategies in *M. ruginodis* and introduces a social supergene in a new ant lineage, adding to the 5 already known.

## Methods

### Overview

To determine if *M. ruginodis* has a novel supergene controlling social organization and queen size, we conducted 4 main analyses. First, we measured queen head width and generated whole-genome sequencing data from 17 queens/gynes to search for genome regions associated with size. Second, we used targeted sequencing to genotype an additional 78 queens to verify our findings. Third, we tested whether the supergene was associated with colony social organization by estimating the number of matrilines within sampled colonies using microsatellite data from workers. And 4th, we used orthology and synteny analyses to assess whether the *M. ruginodis* supergene shares gene content with other known ant supergenes.

### Nomenclature

We follow the British tradition of referring to wingless adult reproductive females as *queens*, and newly hatched and winged reproductives as *gynes*. Moreover, with *single-queen* and *multiple-queen* colonies we refer only to the observed number of queens. Fast queen turnover, colony-founding by budding with concurrent queen dispersal, and failure to sample all queens, may result in the observed queen number not corresponding to the true number of breeding queens in the colony (see above). We therefore refer to colonies where all workers are offspring of a single queen as *monogynous* and those with multiple reproducing queens as *polygynous*.

## Whole-genome Sequencing Data: Sampling and Analyses

### Field Work, Morphometrics, and WGS Sequencing

We collected 31 *M. ruginodis* colonies from 2 separate locations (>10 km apart), both in the vicinity of Tvärminne Zoological station, Hanko, SW-Finland, in 2022 to 2024 (Table S1). The Hanko area is characterized by dry pine forest, where *M. ruginodis* workers build their colonies in a thin layer of vegetation above the sandy bottom. This allows excavation of entire colonies, a method that rarely lets queens escape collection (Seppä 1994; Wolf et al. 2018). We manually sorted each colony in the lab and collected all queens and gynes, as well as a subset of workers.

From the 7 colonies collected in 2023 (Table S1), we decapitated all queens (*n* = 11) and gynes (*n* = 10) with a sterilized scalpel and photographed the head from a dorsal view using a microscope camera. We used a cell counting chamber (a glass plate with a 0.02 mm grid) as a scale and extracted precise measurements of the head width (maximum width directly above the eyes) using ImageJ2 (Rueden et al. 2017) (Table S1). A cut-off value of 1.06 mm has been used previously to distinguish macrogynes and microgynes (Elmes 1991; Wolf et al. 2018). As many of the measured individuals were close to this, we categorized queens and gynes >1.08 mm as macrogynes, <1.04 mm as microgynes, and 1.04 to 1.08 mm as intermediates. We flash froze the bodies of the queens/gynes, along with workers from the same colonies, in liquid nitrogen and stored them in −80 °C. We extracted DNA from all queens (*n* = 11/11) and a subset of the gynes (*n* = 6/10) using DNeasy Blood & Tissue Kit (QIAGEN), followed by whole-genome sequencing on an Illumina NovaSeq X Plus (PE150) platform with a Plant and animal whole genome library preparation (350 bp). From the remaining 24 colonies collected in 2024 (Table S1), all captured queens (*n* = 78) were stored in 96% ethanol until decapitation and morphometric photography (following the same protocol as above), and then stored in −20 °C along with a subset of workers from each colony.

We also whole-genome sequenced 18 *M. ruginodis* workers, each collected from a different colony in the Hanko area in 2022 (Table S1), using the same DNA extraction and sequencing protocols as described above.

### Chromosome-Level Pseudo-Assembly of the *M. rubra* Reference Genome

The genome of *M. rubra*, a sister-species to *M. ruginodis*, was recently assembled as part of the Global Ant Genomics Alliance (GAGA) consortium initiative to generate reference genomes for lineage-representatives of each ant genus (Vizueta et al. 2024). To increase contiguity of this scaffold-level assembly (N50: 1.4 Mb; 477.0 Mb; Table S2), we corrected and scaffolded the genome sequences using the chromosome-level reference genome of *Solenopsis invicta* (N50: 26.2 Mb, total length: 378.1 Mb; Table S2). The 2 species have an estimated divergence time of ∼100 Mya (Prebus and Rabeling 2024).

Specifically, the PacBio reads underlying the *M. rubra* reference genome (SRR31046740, SRR31046742, SRR31046743) were transformed to FASTQ format using bam2fastq from the PacBio BAM toolkit v3.0.0 (https://github.com/PacificBiosciences/pbtk), and corrected using canu v2.2 (Koren et al. 2017). The corrected reads had 27.09 times sequence depth (Table S3). We used ragtag correct v2.1.0 (Alonge et al. 2022) to break scaffolds in the *M. rubra* genome at potential misassembly sites using *S. invicta* as a target genome. To avoid breaking scaffolds in incorrect places and within genic regions, we provided the corrected PacBio reads as supporting evidence as well as the *M. rubra* gene annotation file (GFF). The corrected reference genome was then scaffolded using *S. invicta* as a target genome. The scaffolded *M. rubra* reference genome had an N50 value of 18.3 Mb and a total length of 477.1 Mb (Table S2). The genome coordinates of the *M. rubra* gene annotation were translated to the new genomes using ragtag update GFF, first from the original to the corrected version, and then from the corrected version to the scaffolded version.

Genome statistics were calculated with QUAST v5.2.0 (Gurevich et al. 2013). We assessed the genome completeness of the original and scaffolded *M. rubra* genome using BUSCO v5.4.7 (Simão et al. 2015) using the hymenoptera_odb10 dataset (eukaryota, 2024-01-08), and found that the scaffolding did not lower the quality of the reference genome (original *M. rubra*: 94.1% complete BUSCOs; *M. rubra* scaffolded: 94.2% complete BUSCOs; Table S4). To visualize the position of the original *M. rubra* scaffolds along chromosome 5 in the chromosome-level pseudo-assembly, we aligned the 2 reference genomes using minimap2 v2.28 (Li 2018). Figure S1 shows the ranges of *M. rubra* scaffolds aligning with >100 kb along chromosome 5 in the pseudo-assembly.

### Alignment and Variant Calling

Whole-genome sequencing data from the 17 *M. ruginodis* queens/gynes and 18 *M. ruginodis* workers (Table S1) were aligned to the scaffolded *M. rubra* reference genome using NextGenMap v0.5.5 (Sedlazeck et al. 2013), sorted with samtools v1.16.1 (Danecek et al. 2021), and deduplicated with picard v2.27.5 (http://broadinstitute.github.io/picard/). We calculated alignment statistics based on the deduplicated BAM files using samtools stats. The sequencing depth for the queens/gynes varied between 16.1x and 21.7x (x̄ = 18.1x), and for the workers between 11.2x and 13.0x (x̄ = 11.8x; Table S5). Variants were called for each sample separately using deepvariant v1.4.0 (Yun et al. 2021), followed by joint variant calling with glnexus v1.4.1 (Yun et al. 2021). We normalized the VCF file with bcftools norm v1.16 (Danecek et al. 2021) and filtered using vcftools v0.1.17 (−min-alleles 2 –max-alleles 2 –minQ 20 –minDP 3 –max-missing 0.6 –remove-filtered-all) (Danecek et al. 2011). We calculated pairwise F_ST_ between macrogynes, microgynes, and intermediates, across 10 kb windows with vcftools.

To visualize genotypes across the supergene region on chromosome 5, we made a rolling window PCA plot by first filtering the original VCF file for this chromosome, and then splitting the new VCF file into separate files for each 50 kb window. These 50 kb VCF files were transformed to PLINK format using plink v2.00a6 (www.cog-genomics.org/plink/2.0/) (Chang et al. 2015), followed by a PCA analysis using plink for each window. PCA axis 1 was plotted along the chromosome using ggplot2 v3.5.1 (Villanueva and Chen 2019) in R v4.4.1 (R Core Team 2022). Heterozygosity across chromosome 5 was calculated with vcftools using the –het option (Table S6). Linkage disequilibrium between SNPs is expected to be constant throughout supergene regions, while rapidly decaying with distance in normally recombining (non-supergene) regions. We therefore calculated decay of linkage disequilibrium for (i) each chromosome (1 to 16), for the (ii) supergene and (iii) non-supergene regions of chromosome 5 using PopLDdecay v3.43 (Zhang et al. 2019). For each resulting file, we used the script Plot_OnePop.pl from the same program to calculate r^2^ values across bins using the default settings. We used values from all chromosomes except chromosome 5 to calculate genome-wide r^2^ values.

### Supergene Haplotype Gene Content

All whole-genome sequenced samples (*n* = 17 queens/gynes and *n* = 18 workers; Table S1) were categorized as AA, AB, or BB genotypes according to which SNP genotypes were most common within the supergene region (see Results). Fixed haplotype-specific within-exon SNPs (heterozygous in AB individuals, while having opposing homozygosity between all AA and BB individuals) were extracted. First, all SNPs that were heterozygotic in all AB individuals were extracted using snpSift v5.1d (Cingolani et al. 2012). Second, we calculated F_ST_ between the AA and BB individuals using vcftools with the –weir-fst-pop option and selected only SNPs with F_ST_ values of 1. Lastly, we extracted the longest transcript from each gene in the GFF file using the AGAT v1.0.0 (Dainat) toolkit and the script agat_sp_keep_longest_isoform.pl, and extracted only the SNPs within CDS regions (*n* = 594 SNPs). Then we used orthofinder v2.5.5 (Emms and Kelly 2019) to find orthologs between selected ant species with high-quality reference genomes and their associated gene annotations (*Atta*, *Solenopsis*, *Pogonomyrmex*, *Myrmica*; Table S2).

### Synteny

We analyzed synteny between 4 lineages where the genome region for supergenes underlying colony social organization is known: *Formica* (Brelsford et al. 2020), *Myrmica* (this study), *Pogonomyrmex* (Errbii et al. 2024), and *Solenopsis* (Yan et al. 2020) (see Tables S2 and S7 for genome and annotation versions, as well as supergene genome regions for each reference genome). We used the GENESPACE v1.3.1 (Lovell et al. 2022) analytical pipeline, which relies on orthofinder to identify orthologs, and MCScanX (Wang et al. 2012) (downloaded from GitHub 26th of September 2024) for visualization of syntenic blocks. Prior to analysis with GENESPACE we filtered each GFF file for the longest transcript for each gene using the script agat_sp_keep_longest_isoform.pl from the AGAT toolkit. The output from GENESPACE was plotted using Circos v0.69-8 (Krzywinski et al. 2009).

## Confirming the Association Between Queen Morph and Supergene With Targeted Genetic Markers

### Supergene Genotyping Through Targeted Sequencing Experiments

Using the 17 whole-genome sequenced *M. ruginodis* queens/gynes (Table S1), we designed diagnostic PCR primers for the supergene, capable of distinguishing between AA, AB, and BB genotypes through a B-specific 2 bp insertion (primer pair “IL16”; Table S8). The same individuals were used to confirm the DNA product accuracy through PCR and Sanger sequencing. The resulting electropherogram files (ab1 format) were phased using the R package sangerseqR v1.40.0 (Hill et al. 2014), using one of the whole-genome sequenced queens with an AA supergene genotype (MrugQ14; Table S1) as a reference. The sequences were trimmed to the region between 50 and 200 bp and aligned using muscle v5.1 (Edgar 2022). The region around the diagnostic 2 bp indel was plotted for all samples using the R package ggmsa v1.10.0 (Zhou et al. 2022), which showed a perfect correlation to the supergene genotype assignment from the whole-genome sequencing data (Table S1; Figure S2).

We then extracted DNA from all remaining queens (*n* = 78; Methods section 1.1; Table S1) using DNeasy Blood & Tissue Kit (QIAGEN), and used the (1) IL16 primers to genotype them for the supergene and (2) the universal mitochondrial primer pair LCO1490 and HCO2198 (Folmer et al. 1994) to confirm the species identity. Details on primer pairs and PCR protocol are provided in Table S8. For the mitochondrial marker, targeting the COI gene, we aligned the sequences using muscle and then trimmed the aligned sequences to the region between 22 and 680 bp. We used the R package ape v5.8 (Paradis et al. 2004) to calculate pairwise sequence distance using the default K80 method between all individuals. The sequence distances were very low between all individuals (ranging between 0 and 0.006), confirming that they are all *M. ruginodis* (Figure S3).

### DNA Microsatellite Genotyping

To confirm the kin structure in single-queen colonies (see above), we investigated the genetic relationship between workers using 5 microsatellite markers (Table S9). All markers were originally designed for other *Myrmica* species (Henrich et al. 2003; Azuma et al. 2005; Zeisset et al. 2005) and also tested directly in *M. ruginodis* (Wolf et al. 2018). DNA was extracted using 6% Chelex and Proteinase K. For details on the PCR protocol for each marker see Wolf et al., (2018). We analyzed 10-11 workers from thirteen single-queen colonies, and 3-8 workers from 5 single-queen colonies (Table S10). As a control, we also investigated worker relationships in 4 multiple-queen colonies (Table S10). Genotypes were predicted for each marker with the GUI program STRyper v1.2.2 (Peccoud 2023). Each peak was manually inspected in STRyper, and incorrect genotype calls were adjusted (Tables S11 to S15 show the original and adjusted genotype calls for each marker; Table S10 shows the final scored genotypes). We tested the genetic relationship among workers by running all 199 samples simultaneously in Colony v2.0.7.1 (Wang 2018). We used the default settings for haplodiploid organisms in the analysis and an allelic dropout rate and false allele rate of 5%. As a small proportion of *M. ruginodis* queens mate multiple times (∼16%; Seppä 1994), we ran the algorithm both with and without polyandry (i.e. maternal polygamy).

## Results and Discussion

### Association Between Queen Size and Novel Supergene Polymorphism

**Fig. 1.**
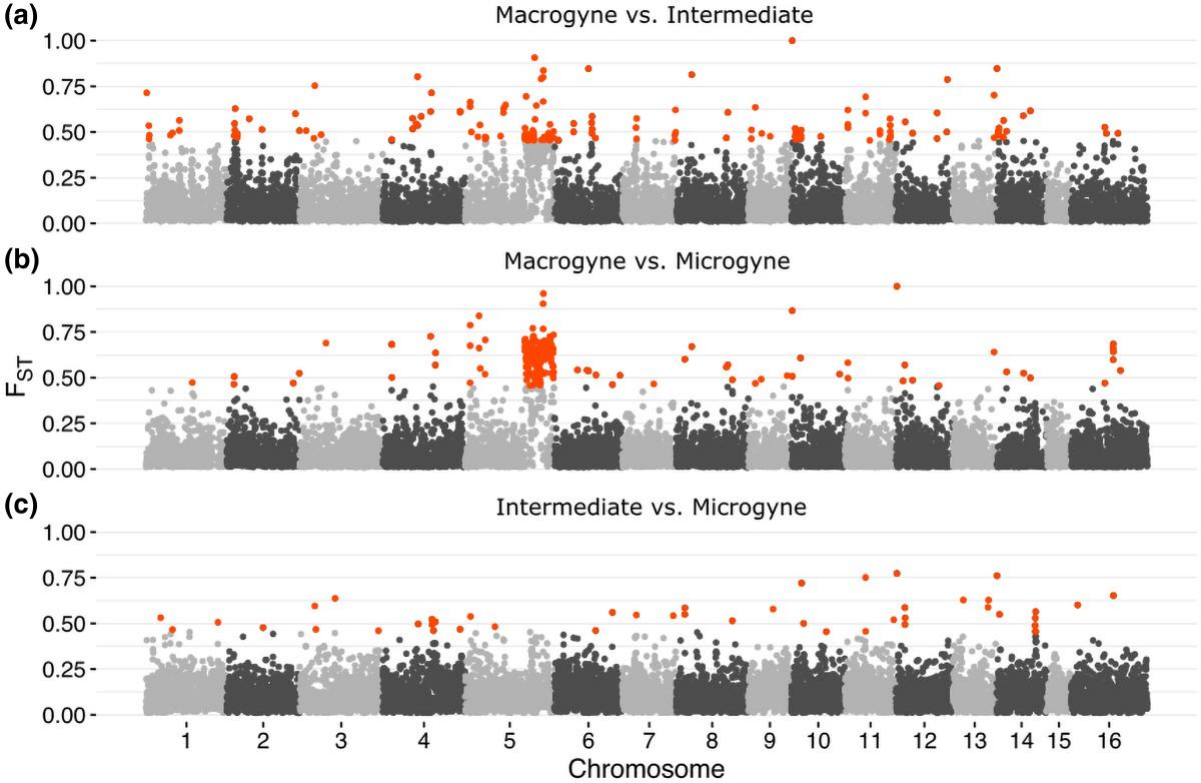
Genome-wide F_ST_ scans across 25 kb windows between the a) macrogynes (*n* = 3; head width >1.08 mm) and intermediates (*n* = 4; head width 1.04 to 1.08), b) macrogynes and microgynes (*n* = 10; head width <1.04), and c) intermediates and microgynes. A 9 Mb region on chromosome 5 was significantly differentiated (values above 0.45, the 95th percentile, are highlighted in red) between macrogynes and microgynes b), somewhat differentiated between macrogynes and intermediates a), but not between intermediates and microgynes c).

To identify genome regions associated with queen size, we aligned genome sequences from the 17 *M. ruginodis* queens/gynes to the pseudo-scaffolded chromosome-level *M. rubra* reference genome. The head sizes ranged between 0.92 and 1.14 mm, thereby categorizing 3 as macrogynes, 5 as intermediates, and 9 as microgynes (Table S1). We searched for local peaks of differentiation between these 3 queen types across all chromosomes using F_ST_, which revealed a highly differentiated region on chromosome 5 spanning 9 Mb (20.2 to 29.2 Mb; Fig. 1). This region was significantly differentiated between macrogynes and microgynes (Fig. 1b), somewhat differentiated between macrogynes and intermediates (Fig. 1a), but not between intermediates and microgynes (Fig. 1c).

**Fig. 2.**
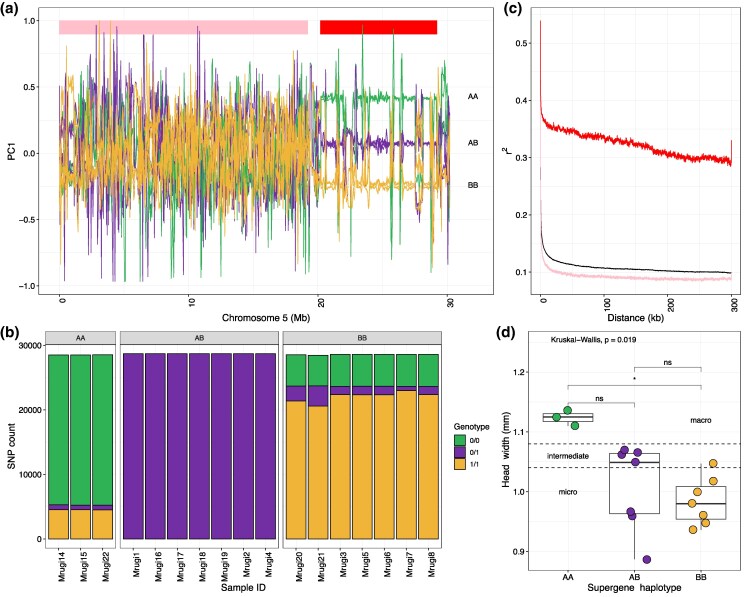
a) Rolling window PCA1 values across chromosome 5 for all queens/gynes (*n* = 17), revealing 3 distinct supergene genotypes (“AA”, “AB”, and “BB”) on chromosome 5 between 20.2 and 29.2 Mb (region marked with a red rectangle). The line colour corresponds to the supergene genotype of each queen/gyne (same as in d). b) Genotype counts for SNPs in the supergene region that are heterozygous in all AB individuals (*n* = 53,817 SNPs). 0/0 = reference allele homozygous, 0/1 = heterozygous, 1/1 = alternative allele homozygous. c) Decay of linkage disequilibrium within the chromosome 5 supergene region (red; region showed in a), the non-supergene region (pink; region showed in a) and a genome-wide estimate for all chromosomes (1 to 16) except chromosome 5 (black). d) Head width measurements for each queen/gyne and supergene category. Data points are colored according to the supergene genotype (see a).

We identified 3 distinct genotypes in the queen morph-differentiated region on chromosome 5, consistent with the expected signature of non-recombining supergene haplotypes, using a 50 kb rolling window PCA (Fig. 2a). Among the queens/gynes in the intermediate PC1 cluster (Fig. 2a; purple), most SNPs in the diverged region were heterozygous (60% to 63%), suggesting that these individuals carry 1 haplotype of each type. We refer to this group as “AB” genotypes. The 2 other PC1 clusters were less heterozygous (13% to 16%) and are interpreted to be homozygous for opposite supergene haplotypes and referred to as “AA” and “BB” (Fig. 2; green and yellow). This grouping is supported by genotype counts of SNPs within this region, where the AA individuals were homozygous for the reference allele for most of the sites where all the AB individuals were heterozygous, while the BB individuals were mostly homozygous for the alternative allele (Fig. 2b). Consistent with the expected patterns from non-recombining supergene haplotypes, the diverged region on chromosome 5 displayed elevated linkage disequilibrium compared to the rest of chromosome 5 and a genome-wide measurement (Fig. 2c). The supergene polymorphism is also present among the 18 *M. ruginodis* workers, as shown by a rolling window PCA (*n* = 7 AA, *n* = 9 AB, *n* = 2 BB) (Figure S4; Table S1).

Previous studies have shown that queen size is a genetically inherited trait in *M. ruginodis* (Wolf 2016). Our findings reveal the genetic architecture underlying this phenotypic divergence. The 3 macrogynes all had the AA genotype, while the intermediates and microgynes had either AB or BB genotypes (Fig. 2d; Table S1). We found a significant association between queen head width and supergene genotype (Kruskal–Wallis, *H* = 7.9173, df = 2, *P*-value = 0.019), with AA genotypes having a higher median head width (1.2 mm) than AB (1.05 mm) and BB genotypes (0.98 mm). After adjusting for multiple tests, only the difference between AA and BB genotypes was statistically significant (Dunn test: z = 2.81; adj. *P* = 0.015; Fig. 2d).

### PCR Genotyping Supports a Link Between Supergene and Queen Size

**Fig. 3.**
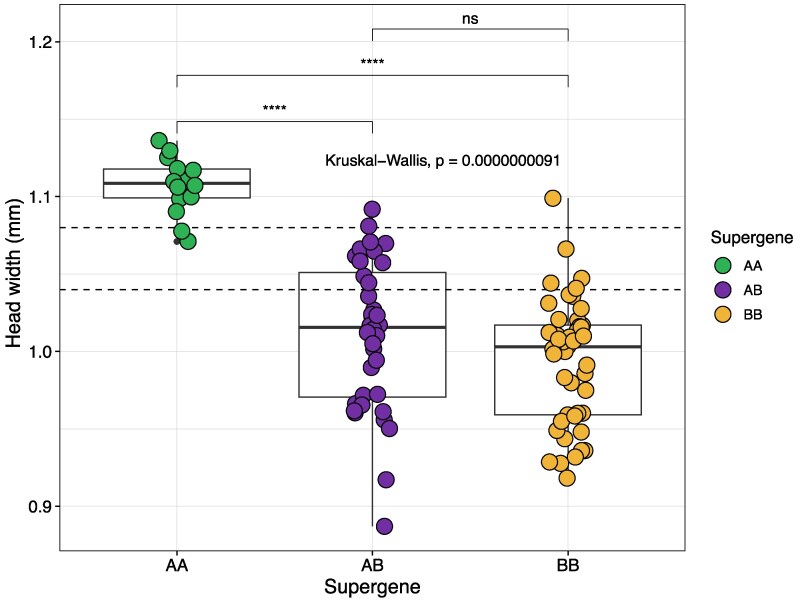
Head width and supergene genotype for the 95 queens/gynes (*n* = 17 WGS sequenced, *n* = 78 PCR genotyped). The dashed lines mark cut-off values between macrogynes, intermediates, and microgynes (as in Fig. 2d).

To verify the association between supergene genotype and queen size, we collected additional *M. ruginodis* queens (*n* = 78; Table S1) and determined their supergene genotype using a diagnostic PCR primer pair (see Methods). Of the 78 *M. ruginodis* queens, 11 were classified as AA homozygotes, 29 as AB heterozygotes, and 38 as BB homozygotes (Table S1; Figures S5 to S7). Again, we found statistical differences in head width between the 3 supergene genotypes (Kruskal-Wallis: H = 37.039, df = 2, *P*-value = <0.0001). The AA genotypes were significantly larger than both AB (Dunn test: z = 4.73; adj. *P*-value < 0.001) and BB (Dunn test: z = 6.08; adj. *P*-value < 0.001) genotypes (Fig. 3). There was, as before (Fig. 2d), no statistical difference between the head sizes of AB and BB genotypes (Dunn test: z = 1.65; adj. *P*-value = 0.10; Fig. 3).

In contrast to the analysis of only the whole-genome sequenced queens and gynes (Fig. 2d), there was some overlap between the head width distribution of the AA genotypes and those of the 2 other groups (Fig. 3). This may be due to error associated with the head width measurements, or due to environmental factors and/or other genetic elements influencing *M. ruginodis* queen size. Similar partial overlaps in supergene-controlled queen size have been found in *Formica cinerea* (Scarparo et al. 2023).

### The Supergene Determines Social Colony Organization

**Fig. 4.**
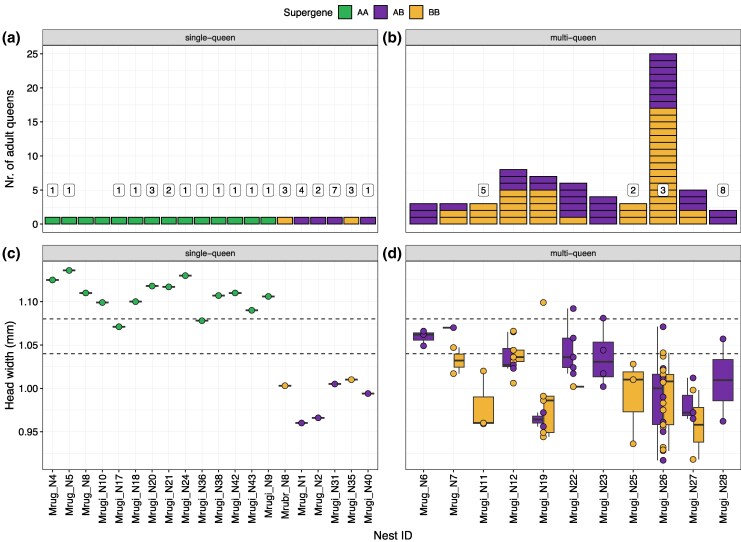
a, b) Number of captured (wingless, i.e. adult) queens and c,d) head width per colony (*n* = 31). The left-side panels a, c) show only colonies with a single queen (*n* = 20) and the right-side panels b, d) show colonies where multiple queens were found (*n* = 11). The numbers within white boxes in panels a) and b) show the number of estimated matrilines based on worker microsatellite data. The dashed lines in c) and d) mark cut-off values between macrogynes, intermediates and microgynes (as in Fig. 2d).

Next, we investigated the association between supergene and colony queen number. The number of captured queens in the 31 colonies ranged between 1 and 25 (x̄ = 2.87; Table S1; Fig. 4), with 20 single-queen and 11 multiple-queen colonies (Fig. 4a, b). Across the 20 single-queen colonies, 14 had AA queens while 6 had AB or BB queens (Fig. 4a). No AA genotypes were found among the 69 queens from the multiple-queen colonies (Fig. 4b). The genotypes in the multiple-queen colonies were either fully AB (*n* = 3), fully BB (*n* = 2) or a mixture (*n* = 6; Fig. 4b). The association between the AA genotype and single-queen nesting was highly significant (Fisher's exact test, *P* = 0.00015). In the single-queen colonies, the 6 non-AA queens were significantly smaller than the AA queens (Dunn test: z = 4.36; adj. *P*-value = <0.0001; Fig. 4c), but of similar size to the non-AA queens in the multiple-queen colonies (Dunn test: z = 1.11; adj. *P*-value = 0.268; Fig. 4c, d). Queen head width appeared bimodally distributed, with queens from single-queen colonies having larger heads that queens from multi-queen colonies, although there was variation within each category (Figure S8).

To further investigate the true sociogenetic structure of single-queen colonies, we clustered worker microsatellite genotypes to matrilines using the software Colony (see Methods). Most of the single-queen AA colonies had only 1 matriline (*n* = 10/12; Fig. 4a; Table S16). However, multiple matrilines were detected in 2 AA colonies (*n* = 2/12; 2 to 3 matrilines; Fig. 4a; Table S16). In contrast, multiple matrilines were detected in almost all non-AA single-queen colonies (*n* = 5/6; 2 to 7 matrilines; Fig. 4a; Table S16). All 4 multiple-queen colonies analyzed as a control had multiple matrilines (*n* = 4/4; 2 to 8 matrilines; Fig. 4b; Table S16). There was a significant association between a single matriline (i.e. monogyny) and queen AA genotype, and between multiple matrilines (i.e. polygyny) and AB or BB queen genotypes (Fisher's exact test, *P* = 0.001905).

There are 2 potential explanations for the non-perfect association between supergene genotype and colony queen number. The first is that monogynous colonies can be headed by small non-AA queens. This seems unlikely, however, as smaller queens are typically less fecund (Brian 1969; Keller 1988; Vargo and Fletcher 1989) and smaller colonies would be at disadvantage when competing with larger ones. Therefore, we argue that the single-queen colonies with non-AA genotypes are most likely polygynous, where additional queens may have died, moved, or escaped sampling. The second explanation is that AA-queens are occasionally found in polygynous colonies. However, our finding of multiple matrilines in some colonies with an AA-queen could also be a result of *serial polygyny*, recruitment of a replacement queen when the resident queen dies (Elmes 1991; Seppä 1994), with a transitory phase where offspring of multiple queens coexist. In *Myrmica*, this likely occurs as a result of the unusually long developmental time of sexual offspring (1 year) (Brian 1983) combined with a relatively short queen life span (∼1.5 years) (Elmes 1980; Seppä 1994; Evans 1995). This often leaves colonies queenless and prone to recruit new, even unrelated, queens (Evans 1996).

One potential issue is that the mutation rate in microsatellite markers is high, and they are prone to genotyping errors. Even though this can be controlled for in the Colony analysis, it may affect sibship assignments even at low frequencies (Wang 2018). It should also be noted that the result of the Colony analysis was partly ambiguous and the statistical support for finding multiple matrilines in single-queen colonies was not always strong (Table S16). In fact, worker genotypes in both single-queen colonies with multiple matrilines detected could also be explained with a single multiply mated queen (Table S10). Future studies using a larger sample of workers and genome sequencing data will reveal the relationship between true monogyny and the AA genotype with higher accuracy.

The number of estimated matrilines in the multi-queen colonies were different from the number of sampled queens in all 4 measured colonies (Fig. 4b). Colonies with more estimated matrilines than queens (e.g. Mrugi_N28; Fig. 2b) could be explained by incomplete sampling, or by recent queen death or migration. Colonies with fewer estimated matrilines (e.g. Mrugi_N26; Fig. 4b) could be explained by too low number of workers analyzed, or if some queens have not yet contributed to the worker force.

### Candidate Genes for Queen Size and/or Colony Social Organization

Supergenes are characterized by low recombination between haplotypes, meaning that genes within these regions are inherited as a single unit. This tight linkage makes it difficult to determine which specific genes contribute to the trait controlled by the supergene. Genes with many haplotype-specific SNPs—variants that are fixed for opposite homozygous alleles in AA and BB individuals, while heterozygous in AB individuals—are more likely than others to play a role in the trait associated with the supergene.

To narrow down potential candidate genes, we counted the number of haplotype-specific SNPs within coding sequence (CDS) regions for each gene within the 9 Mb supergene region (Table S17). The 2 genes with the most haplotype-specific SNPs were *ABCA13* (“ATP-binding cassette, sub-family A”; *n* = 57) and *Twitchin* (*n* = 45; Table S17). In *Drosophila*, knockdown of *ABCA13* has been shown to affect social behavior, leading to an increased distance between individuals (Ueoka et al. 2018). *Twitchin* plays a regulatory role in muscle contraction, and according to 1 study, insect flight muscles (Ayme-Southgate et al. 1991). Relevantly, in 2 obligately polygynous species of the *Formica rufa* group, one of the few remaining genes from a polygyny-associated supergene is *Zasp52*, a flight-muscle gene (Liao et al. 2016; Sigeman et al. 2024). Given that dispersal strategy is a key factor in alternative reproductive strategies in ants, differences in genes affecting flight capability would not be unexpected. However, additional data are needed to confirm the specific roles of these genes in shaping dispersal and social structure.

### Minimal Shared Synteny With Other Ant Social Supergenes

Social supergenes controlling queen number have now been found in 6 lineages of ants; *Cataglyphis*, *Formica*, *Leptothorax*, *Myrmica*, *Pogonomyrmex*, and *Solenopsis* (Wang et al. 2013; Purcell et al. 2014; Braim 2015; Errbii et al. 2024; Lajmi et al. 2024) (this study). Previous studies have reported no gene overlap between the supergenes of *Formica*, *Solenopsis*, and *Pogonomyrmex* (Brelsford et al. 2020; Errbii et al. 2024), suggesting that they arose through independent evolutionary processes from different starting chromosomes. A recent study showed that the supergene of *Cataglyphis* shares synteny with the *Solenopsis* supergene (Lajmi et al. 2024). However, since *Cataglyphis* is more closely related to *Formica* than *Solenopsis*, the authors proposed that the supergene arose independently of the 1 in *Solenopsis* (Lajmi et al. 2024). For the last supergene, detected in *Leptothorax* (Braim 2015), the size and gene content are not yet well characterized (Kay et al. 2022).

**Fig. 5.**
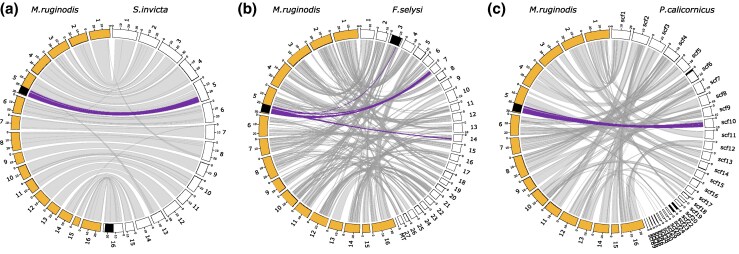
Synteny plots between *M. ruginodis* and a) *S. invicta*, b) *F. selysi*, and c) *P. californicus*. *M. ruginodis* chromosomes are in yellow (filled), and in white (unfilled) for the comparison species. Social supergene regions in each species are highlighted in black. Gray links show genome-wide syntenic regions between reference genomes. Purple links highlight syntenic gene pairs within 10 kb genome windows in the *M. ruginodis* reference genome containing haplotype-specific SNPs. There is no gene overlap between the supergene regions of *M. ruginodis* and a) *S. invicta*, or c) *P. californicus*. Between *M. ruginodis* and b) *F. selysi*, however, a small fraction of genes (*n* = 12; Table S18) are shared between the supergene regions. There is, however, no gene content shared between the Myrmica supergene and *F. selysi* chromosome 9, which is homologous to the chromosome carrying the queen-size controlling supergene in polygynous *F. cinerea* colonies (Scarparo et al. 2023). The *C. niger* supergene shares synteny with the *S. invicta* supergene region on chromosome 16 (Lajmi et al. 2024), but no precise supergene genome coordinates are available for the currently published reference genomes of this species.

Synteny analyses of the *Myrmica* supergene against those of *Solenopsis*, *Formica*, and *Pogonomyrmex* revealed very little gene overlap (Fig. 5). Only 12 *Myrmica* genes are shared between any of the other supergene regions, between *Myrmica* and *Formica* (Fig. 5b; Table S18). Three of these shared genes had haplotype-specific SNPs within their CDS regions, although few: Mrub_g18249_i1 (*n* = 1), Mrub_g05933_i1 (*n* = 1), and Mrub_g08367_i1 (*n* = 3; Table S18). This suggests that the *Myrmica* supergene evolved independently from the others. Note that the *M. rubra* reference genome was used in this analysis as there is no *M. ruginodis* reference genome available. In addition, the pseudo-scaffolding based on the *S. invicta* reference genome (see Methods) likely introduced further disruptions of collinearity. As a result, the gene order within the *M. ruginodis* supergene region may differ from the one shown here. Future studies using a high-quality reference genome from *M. ruginodis* will better characterize the structure and gene order of the supergene.

## Conclusion

We demonstrate that a 9 Mb supergene is associated with queen size and colony social organization in *M. ruginodis*. One genotype of the supergene (AA) is found exclusively in large queens in monogynous colonies. The 2 others (AB, BB) are found in smaller queens in polygynous colonies. This suggests that colony social organization and queen morph are genetically controlled by this supergene in *M. ruginodis*. Through comparative genomics, we show that the novel supergene does not share synteny with other ant supergenes, except for 12 genes that are shared between the *Myrmica* and *Formica* supergenes. The high frequency of all supergene genotypes (AA, AB, and BB) suggests that no combination carries severe fitness consequences. Our findings align with the patterns found in previous research on the queen-size dimorphic ant *M. ruginodis*, while providing more accurate information on the genetic mechanism maintaining distinct social organization in the 2 morphs.

## Supplementary Material

msaf255_Supplementary_Data

## Data Availability

All whole-genome sequencing data underlying this article is available in the NCBI Sequence Read Archive (SRA) under BioProject accession no. PRJNA1331534. All code used in this study is available at GitHub: https://github.com/hsigeman/M_ruginodis_MBE_2025.
