# Supplementary material for: A Novel Supergene Controls Queen Size and Colony Social Organization in the Ant Myrmica ruginodis
Source: Mol Biol Evol. 2025 Oct 13;42(11):msaf255. doi: 10.1093/molbev/msaf255 (PMC12572780; doi:10.1093/molbev/msaf255)
Supplement: msaf255_Supplementary_Data [file msaf255_supplementary_data.zip › Supplementary Figures.pdf]

Supplementary Figures S1-S8

A novel supergene controls queen size and colony social organization in the ant *Myrmica ruginodis*

Hanna Sigeman[1,2], Perttu Seppä[3], Philip A. Downing[2], Matthew T. Webster[1], Heikki Helanterä[2,4], and Lumi Viljakainen[2]

[1]Department of Medical Biochemistry and Microbiology, SciLifeLab, Uppsala University, Sweden
[2]Ecology and Genetics Research Unit, University of Oulu, Finland
[3]Organismal and Evolutionary Biology Research Programme, University of Helsinki, Finland
[4]Tvärminne Zoological Station, University of Helsinki, Finland

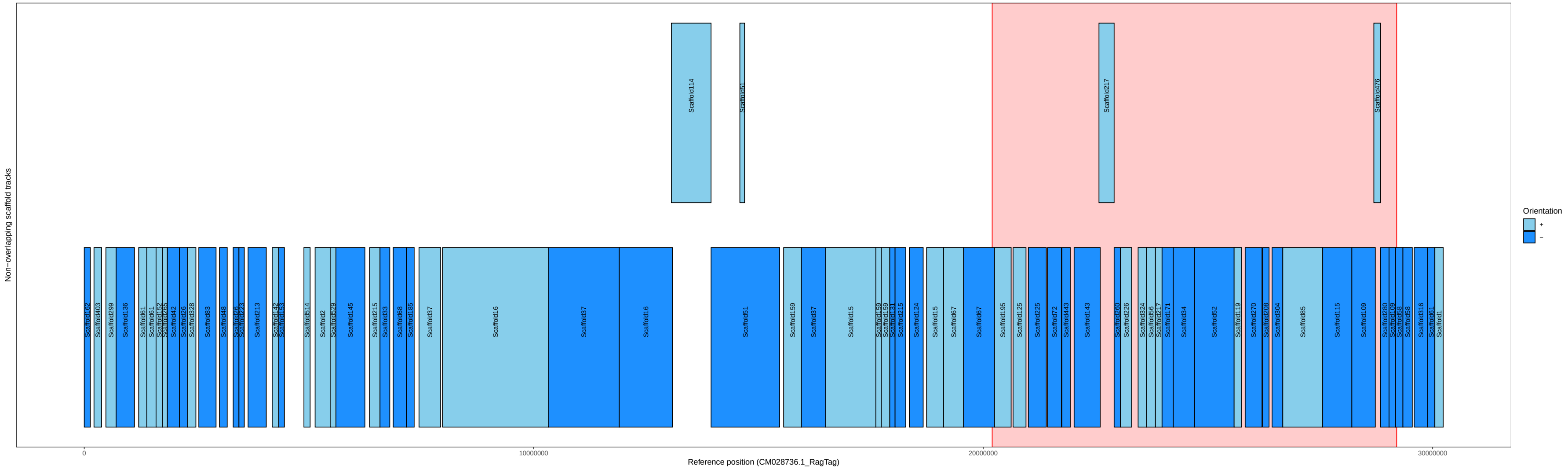

Figure S1: Scaffold alignment ranges from the original *M. rubra* assembly mapped onto chromosome 5 of the chromosome-level pseudo-assembly (see Methods). Only ranges mapping with >100 kb are shown here. Scaffolds are coloured by alignment orientation (+ = lightblue, - = darkblue), and plotted in non-overlapping tracks for clarity. The supergene region (20.2-29.2 Mb) is marked with a red background colour.

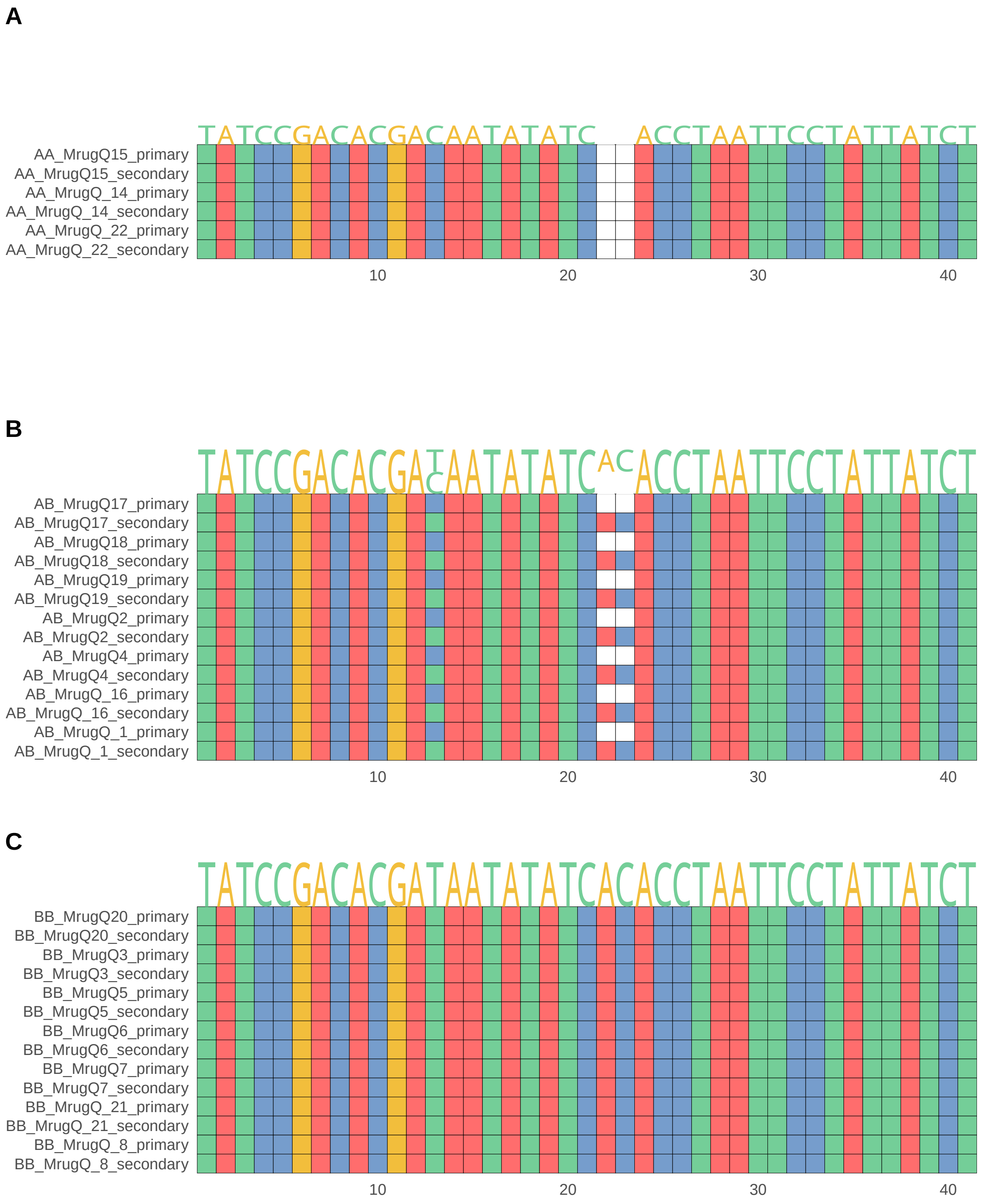

Figure S2: DNA motifs from the supergene haplotype diagnostic primer pair IL16 for 17 whole-genome sequenced queens/gynes. For each individual, electropherogram data from Sanger sequencing were phased into a 'primary' and 'secondary' sequence. The supergene genotype can be inferred through a B-specific 2bp insertion (position 22-23). The queens/gynes are grouped according to their supergene genotype: (**A**) AA, (**B**) AB,(**C**) BB (Table S1).

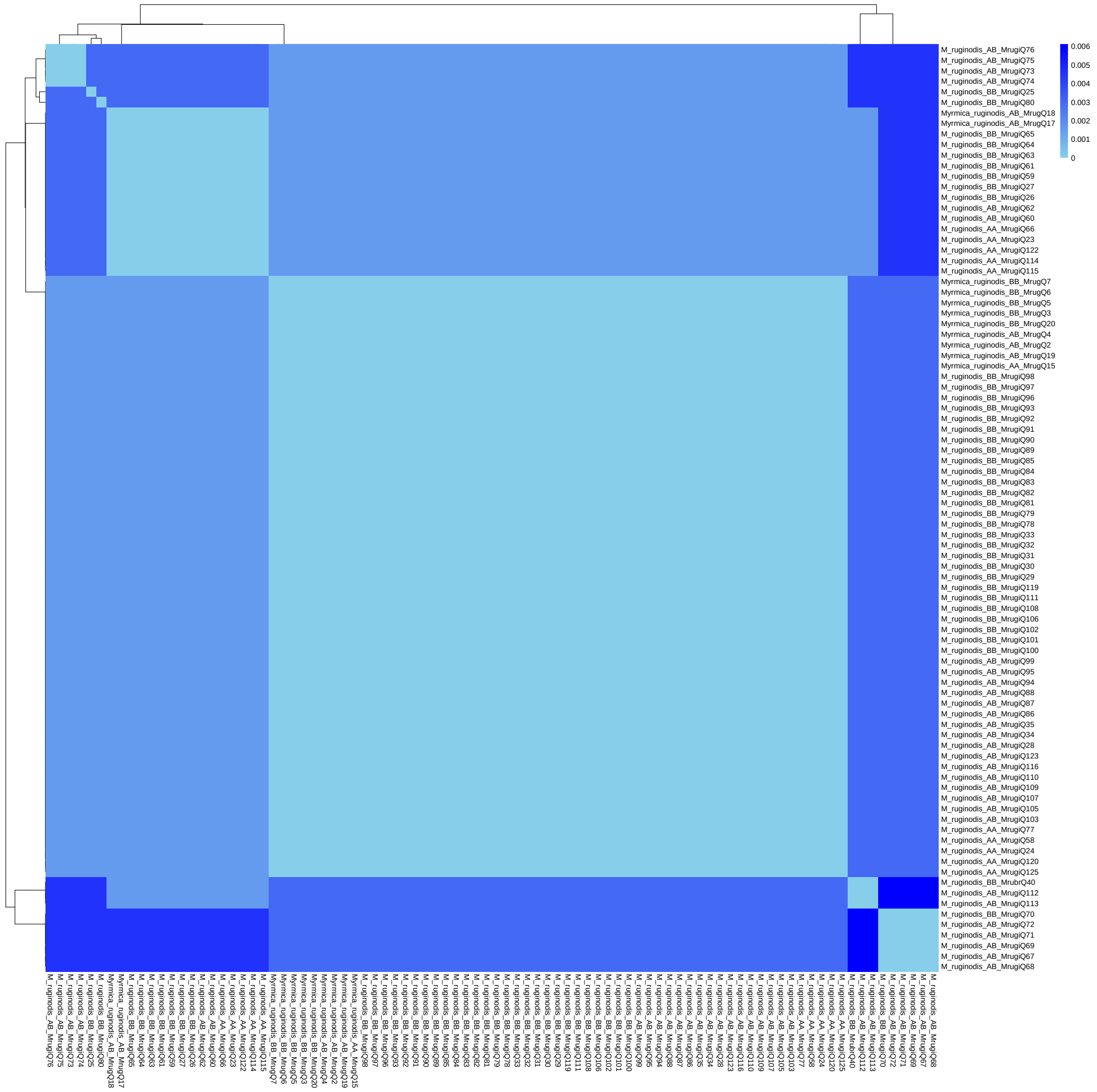

Figure S3: Heatmap showing pairwise sequence distances between COI sequences from *M. ruginodis* queens/gynes. The sequence distances were small between all individuals, ranging between 0 and 0.006, confirming that all individuals belong to the same species.

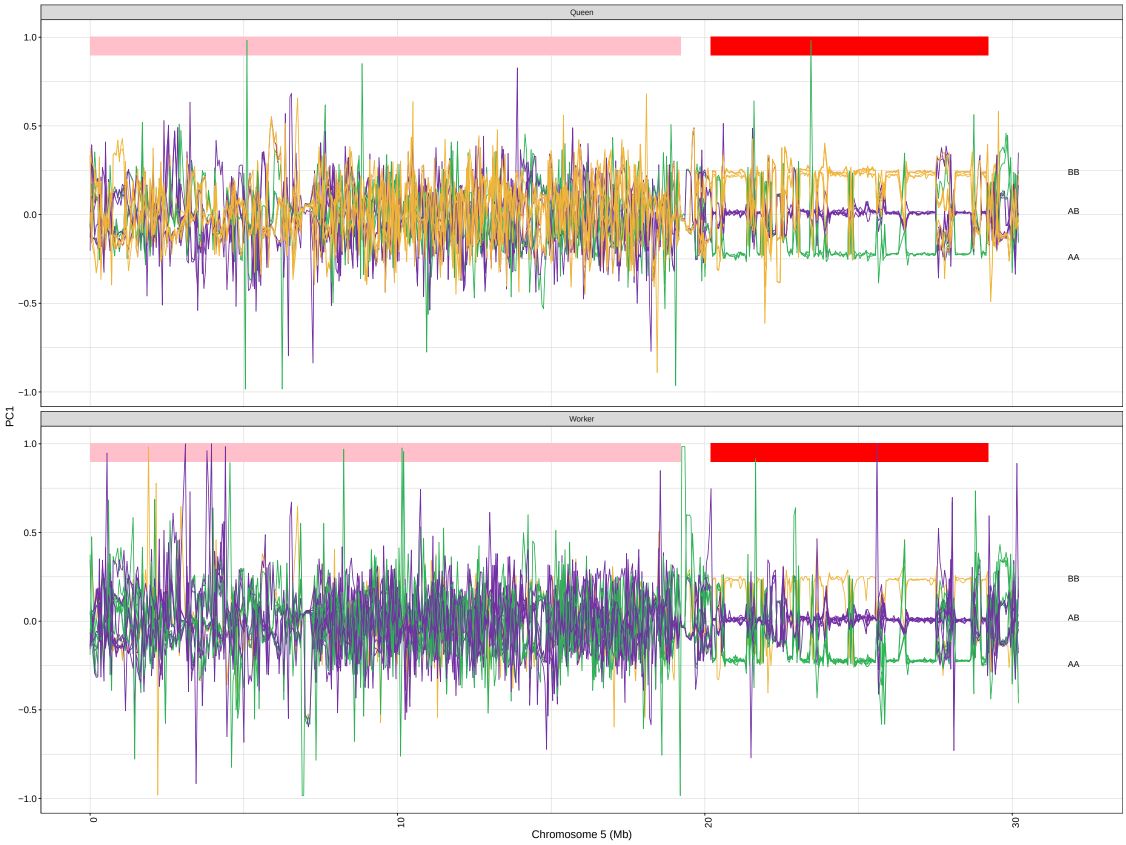

Figure S4: Rolling PCA plot across chromosome 5, showing the three supergene genotypes AA, AB, and BB. The upper panel show data for the 17 whole-genome sequenced *M. ruginodis* queens/gynes (same individuals as Figure 2A) and the bottom panel show data from 18 *M. ruginodis* workers. See Table S1 for sample details.

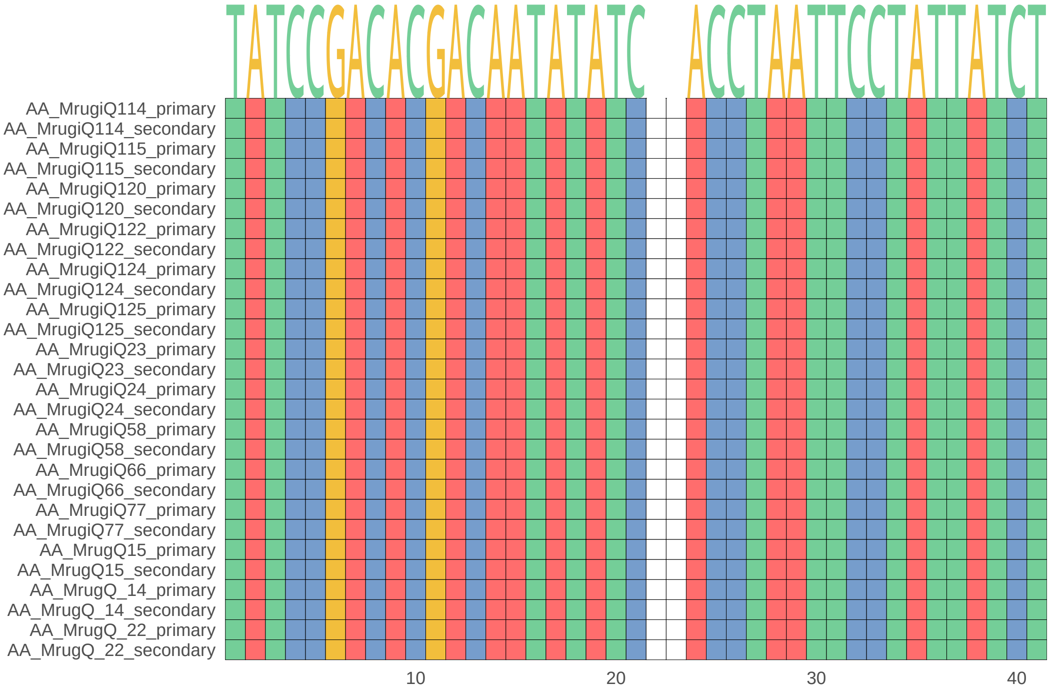

Figure S5: All additional (non-whole genome sequenced) AA queens genotyped using the IL16 supergene diagnostic PCR primers. See Table S1 for sample details.

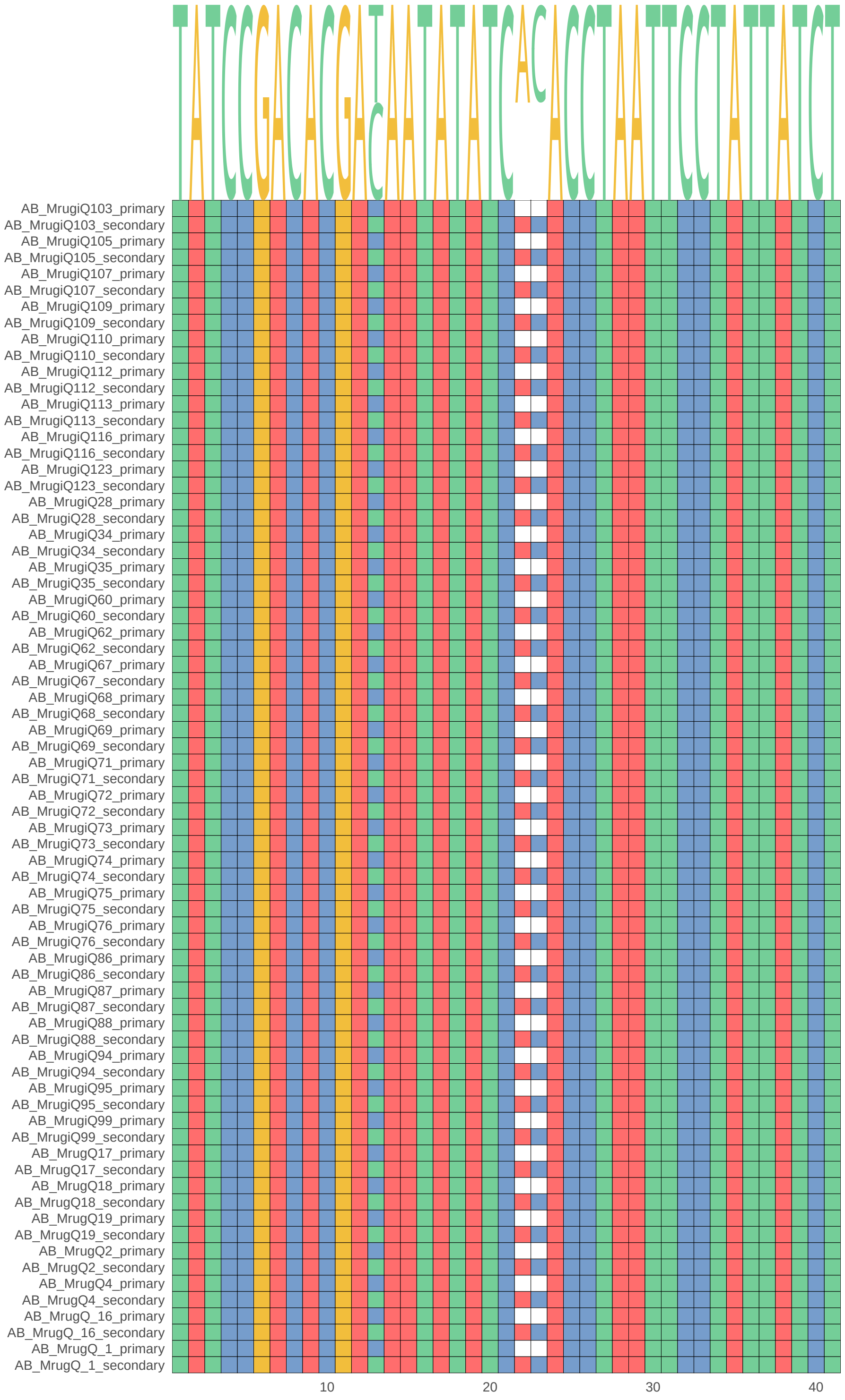

Figure S6: All additional (non-whole genome sequenced) AB queens genotyped using the IL16 supergene diagnostic PCR primers. See Table S1 for sample details.

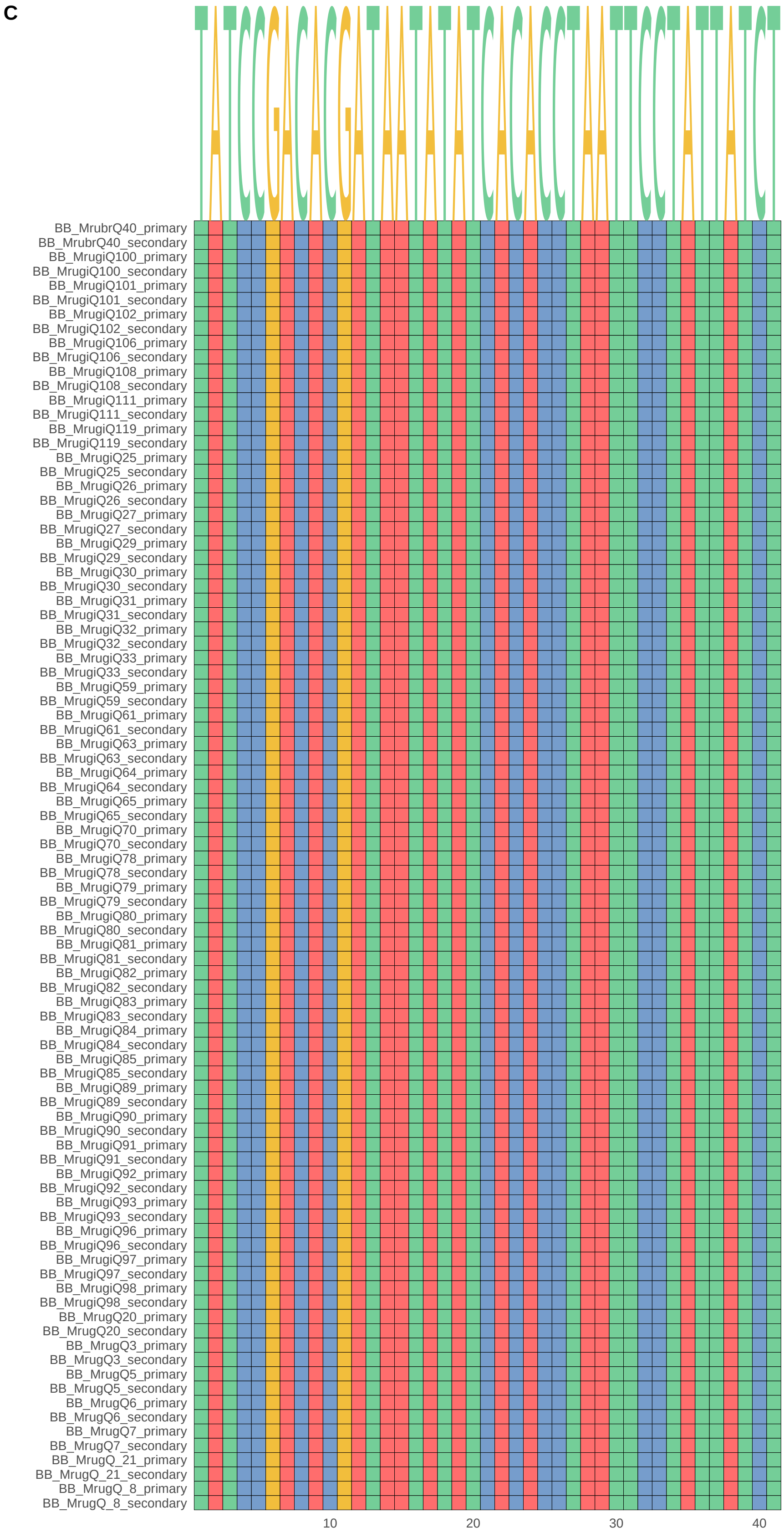

Figure S7: All additional (non-whole genome sequenced) BB queens genotyped using the IL16 supergene diagnostic PCR primers. See Table S1 for sample details.

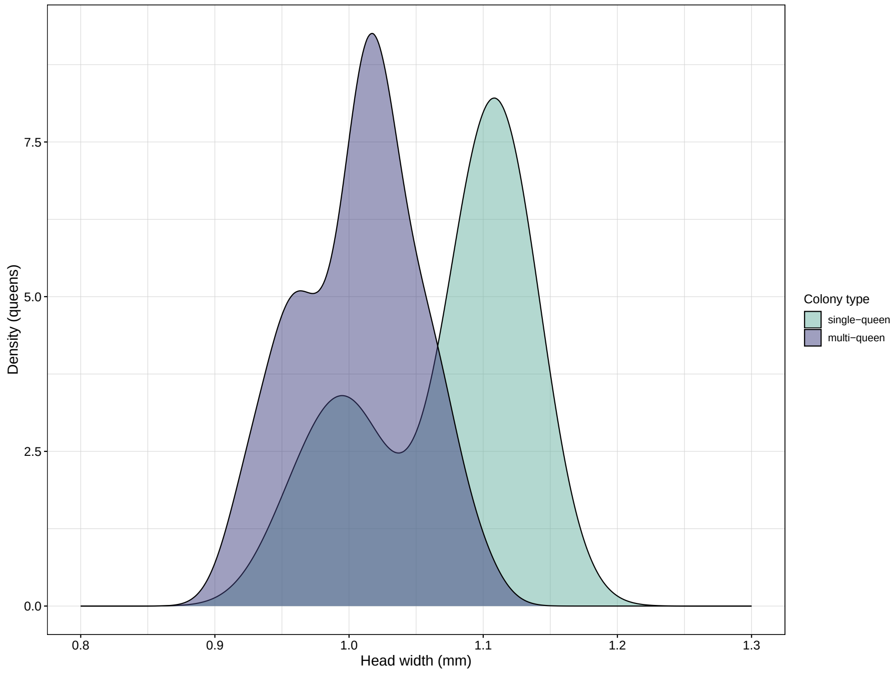

Figure S8: Density plot showing head width (mm) for queens (n = 89) captured from single-queen colonies and multi-queen colonies.
